# Provider Self-Disclosure During the Childhood Vaccine Discussion

**DOI:** 10.1177/0009922818817828

**Published:** 2018-12-05

**Authors:** Katherine Lepere, Nicole Etsekson, Douglas J. Opel

**Affiliations:** 1 Treuman Katz Center for Pediatric Bioethics, Seattle, WA, USA; 2 Seattle Children’s Research Institute, Seattle, WA, USA; 3 University of Washington, Seattle, WA, USA

## Introduction

Provider self-disclosure in medicine is controversial. While some have urged self-disclosure be used cautiously, if at all,^1^ others have argued that a cautious approach to self-disclosure can undermine the ability to leverage its potential benefits.^2^ Though indiscriminate or gratuitous use of self-disclosure can be detrimental, judicious use of self-disclosure can nurture trust and rapport with patients. Studies are inconclusive, however, regarding the impact of provider self-disclosure on patient satisfaction^3–6^ or compliance with medical recommendations.^7,8^

Despite this controversy, provider self-disclosure has become a communication strategy of interest in the pediatric vaccination setting. Pediatric providers report that they disclose vaccinating their own children to vaccine-hesitant parents (VHPs)^9,10^ and that they believe this self-disclosure strategy promotes parental acceptance of vaccines.^9–11^ The Centers for Disease Control and Prevention also encourages providers to use personal anecdotes in communication with VHPs.^12^

Our understanding of how provider self-disclosure manifests during conversations with parents about childhood vaccines, however, is only nascent. For instance, previous studies in the childhood vaccination setting have primarily focused on the specific behavior of providers referring to their own vaccine decision making.^8,9^ It is unknown whether providers use other self-disclosure strategies with parents to support vaccination and whether provider use of self-disclosure is more frequent with certain types of parents or visits in the childhood vaccine setting. The objective of this study was to characterize the use of provider self-disclosure to support vaccination during child health supervision visits.

## Methods

### Study Design

We performed a secondary analysis of videotaped health supervision visits collected as part of a cross-sectional observational study conducted from 2011 to 2014 in order to characterize parent-provider vaccine communication. This study was approved by the Seattle Children’s Institutional Review Board, and all participants provided written informed consent. Methods for this observational study have been described elsewhere.^13–15^ In brief, 23 pediatric providers (pediatricians and pediatric nurse practitioners) from 16 primary care clinics in the Seattle area participated in the study. Participating clinics were from diverse settings and included university-based, multispecialty group, suburban private, community hospital-based, and urban private pediatric clinics. Parents were eligible to participate if they were English speaking, ≥18 years old, and had a 1- to 19-month-old child being seen for a health supervision visit by a participating provider. Prior to the visit, parents completed the previously validated Parent Attitudes about Childhood Vaccines (PACV) survey to determine their vaccine hesitancy status.^16–18^ VHPs, defined as parents who scored ≥50 on the 100-point PACV, were oversampled. After the visit, parents completed an 11-item survey that included questions about parent age, income, marital status, race/ethnicity, gender, number of children in their household, and whether this was their first vaccine discussion with their child’s provider.

The study was described to parents and providers generally as one that sought to understand more about parent-provider communication during well-child check-ups. All health supervision visits were videotaped. Videotapes were edited to contain only the vaccine discussion and subsequently transcribed. The final sample contained 213 videotaped health supervision visits.

### Analysis

#### Coding.

For the current study, we developed a preliminary coding scheme based on existing literature about provider vaccine communication^9^ and self-disclosure practices.^1,19^ Using a subset of transcripts of videotaped encounters (N = 10), 2 investigators (KL and NE) revised this preliminary coding scheme by including additional types of observed self-disclosure behaviors providers used to support childhood vaccination. This revised coding scheme was then discussed and refined with a third investigator (DJO). The final coding scheme (see the appendix) contained 2 types of self-disclosure designed to support vaccination: personal self-disclosure (PSD) and clinical experience self-disclosure (CSD). PSD was defined as a statement by the provider related to himself/herself, his/her immediate family, or friends about vaccine-preventable diseases or vaccine decision-making that supported vaccination (eg, “I got my flu shot this year”). CSD was defined as a statement by the provider about his/her interaction with other patients or health care professionals related to vaccine-preventable diseases or vaccine decision making who supported vaccination (eg, “We have lots of families who do vaccines here”). Disclosures unrelated to vaccination were not included. The coding scheme also included whether each PSD or CSD statement was made in response to a parent’s question or said spontaneously by the provider without parent prompting.

Two investigators (KL and NE) subsequently coded transcripts in sets of 10 independently, including those used to develop the coding scheme. After each set, the investigators met to compare coding results, measure intercoder reliability, and discuss discrepancies with a third investigator (DJO). This process was repeated until intercoder reliability was adequate (*k* > 0.7). One investigator (KL) coded all remaining transcripts and a second investigator (NE) coded every 10th transcript to monitor for potential drifting and ensure that *k* remained above 0.7.

We used descriptive statistics to determine the frequency of self-disclosure types observed in the study sample. We used the χ^2^ test to determine the association of self-disclosure use with parent demographics, visit characteristics, and parent PACV score.

## Results

In our sample of 213 videotaped health supervision visits, 56 (26%) visits included provider use of PSD or CSD. These visits involved 17 (74%) of the 23 participating providers from 10 different participating clinics. Parents in these 56 visits that included PSD or CSD were predominately mothers who were married, white, ≥30 years of age, and had a household income of >$75 000 (Table 1).

Compared with visits in which providers did not use PSD or CSD, visits in which providers used PSD or CSD had a higher proportion of parents with one child in the household (66% vs 49%, *P* = .04) and parents who were having a first-time vaccine discussion with their provider (31% vs 15%; *P* = .01).

Among the 56 visits in which PSD or CSD was used, there were a total of 91 unique instances of PSD or CSD and a mean number of 1.3 (range = 1–7) PSD or CSD instances per videotaped encounter (Table 2).

Overall, the most frequent PSD type used was “provider states he/she has vaccinated (or would vaccinate) his/her own child” (N = 26), and the most frequent CSD type used was “provider states his/her other patients are vaccinated” (N = 26). There were 18 visits (32%) in which both PSD and CSD were used, 19 visits (34%) in which only PSD was used, and 19 visits (34%) in which only CSD was used. A higher proportion of visits included CSD when PSD was (vs was not) used (49% vs 11%; *P* < .001).

In all but 4 of the 91 (4%) unique instances of PSD or CSD, the provider used PSD or CSD spontaneously without the parent prompting the provider for the information with a question. Provider use of PSD was not associated with any parent demographics or visit characteristics. However, providers used CSD in a higher proportion of visits involving VHPs compared with non-VHPs (20% vs 9%, respectively; *P* = .05) and in visits with parents who had 1 child in the household compared with those with ≥2 children in the household (24% vs 10%; *P* = .01).

## Discussion

To our knowledge, this is the first study to assess provider self-disclosure during the childhood vaccine discussion using direct observation methods. We found that self-disclosure designed to support vaccination was used in 26% of visits, which is within the range reported in other medical settings for the proportion of encounters in which providers generally use self-disclosure (9% to 34%).^1,3,5^ We also found that the most frequently used PSD and CSD types involved a disclosure of the act of vaccination rather than a disclosure of the negative consequences of forgoing vaccination. This indicates that providers may be primarily utilizing self-disclosure as an appeal to vaccination as the social norm.^20^

We also found that the majority of visits in which providers used any type of self-disclosure to promote vaccination were with parents having their first vaccine discussion and those with one child in the household. When specifically looking at self-disclosure type, PSD use was not associated with any parent demographics or visit characteristics, but CSD was used with a higher proportion of VHPs and parents who only had one child in their household. Taken together, this data suggest that providers may view self-disclosure, and particularly CSD, to be beneficial in vaccine discussions with first-time parents and those that are new to their practice.

This study has several limitations. It was conducted in a single geographic region with a relatively small sample size and therefore has limited generalizability. In addition, we were unable to determine whether provider use of self-disclosure to support vaccination was associated with increased parental acceptance of vaccines. Replication of this study with a larger, more diverse sample is necessary to answer these important questions.

## Conclusion

Overall, pediatric providers use self-disclosure to promote childhood vaccination infrequently. However, when providers do use self-disclosure, this most often manifests as providers stating that they have vaccinated their own children. Providers are more likely to use self-disclosure during conversations with parents who they have not previously discussed vaccines, and with parents who only have one child in the household.

## Figures and Tables

**Table 1.** Demographics of Study Population^a^.

	Overall (N = 213), n (%)	Visit with PSD or CSD (N = 56), n (%)	Visit without PSD or CSD (N = 157), n (%)	*P*
Relationship to child^b^				
Mother	172 (90%)	47 (89%)	125 (91%)	.69
Parent age (years)^b^				
≥30	142 (74%)	38 (72%)	l04 (75%)	.60
Parent’s marital status^b^				
Single, separated, widowed, or divorced	15 (8%)	4 (8%)	ll (8%)	>.99
Married or living with a partner	176 (92%)	49 (92%)	l27 (92%)	
Parent education^b^				
High school graduate/GED or less	17 (9%)	4 (8%)	13 (9%)	.78
Some college/2-year degree or more	173 (91%)	49 (92%)	124 (91%)	
Household income^b^				
>$75 000	106 (57%)	29 (56%)	77 (57%)	.83
Parent race/ethnicity^b^				
White	148 (79%)	43 (83%)	l05 (77%)	.41
Black or African American	6 (3%)	0 (0%)	6 (4%)	.l9
Hispanic/Latino	2 (1%)	0 (0%)	2 (1%)	>.99
Asian	18 (10%)	5 (10%)	13 (10%)	.99
Native Hawaiian or other Pacific Islander	2 (1%)	0 (0%)	2 (1%)	>.99
American Indian or Alaska Native	0 (0%)	0 (0%)	0 (0%)	—
More than 1 race	12 (6%)	4 (8%)	8 (6%)	.74
Number of children in household^b^				
1	103 (54%)	35 (66%)	68 (49%)	.04
≥2	88 (46%)	18 (34%)	70 (51%)	
First vaccine discussion^b^	32 (l9%)	15 (31%)	17 (l5%)	.01
Vaccine-hesitant parent	157 (74%)	45 (80%)	112 (71%)	.l9

Abbreviations: PSD, personal self-disclosure; CSD, clinical experience self-disclosure.

a Comparison of parents exposed to PSD or CSD versus those not exposed to PSD or CSD using Pearson χ^2^ test, or where cell sizes <5, Fisher’s exact test.

b Numbers do not equal total N because of missing data.

**Table 2.** Instances of Self-Disclosure by Type in the 56 Visits (N = 91).

Personal Self-Disclosure Type	N (%)
1. Provider states she/he has vaccinated (or would vaccinate) his/her own children	26 (56%)
2. Provider states that she/he has been vaccinated	7 (15%)
3. Provider states that his/her friend or family member (other than child) has been vaccinated	3 (7%)
4. Provider states that she/he has had a vaccine preventable disease	3 (7%)
5. Provider states that his/her friend or family member has had a vaccine preventable disease	6 (13%)
6. Other	1 (2%)
Total	46
Clinical Experience Self-Disclosure Type	N (%)
1. Statement about other patients the provider or his/her colleague vaccinated	26 (58%)
2. Statement about other patients the provider or his/her colleague cared for with vaccine preventable disease	16 (36%)
3. Other	3 (6%)
Total	45

## Appendix

**Table:** Coding Scheme.

Personal Experience Self-Disclosure Type	Example
1. Provider states she/he has vaccinated (or would vaccinate) his/her own children.	“I felt really comfortable vaccinating my own kids.”
2. Provider states that she/he has been vaccinated.	“I got my flu shot this year.”
3. Provider states that his/her friend or family member (other than child) has been vaccinated.	“My brother got the DPT vaccine. It made him really uncomfortable. That vaccine is different than the DTaP that we’re giving your baby.”
4. Provider states that she/he has had a vaccine preventable disease.	“I remember I was one of those people who had like 200 pox and high fevers.”
5. Provider states that his/her friend or family member has had a vaccine preventable disease.	“My sister got really sick with chicken pox when we were kids.”
6. Other	
Clinical Experience Self-Disclosure Type	Example
1. Statement about other patients the provider or his/her colleague vaccinated	“We have lots of families who do vaccines here …”
2. Statement about other patients the provider or his/her colleague cared for with vaccine preventable disease.	“I’ve already diagnosed about, it’s been about 2 cases of whooping cough in the last 3 weeks.”
3. Other	
